# Iodine status in pregnant women and infants in Finland

**DOI:** 10.1007/s00394-022-02852-9

**Published:** 2022-03-19

**Authors:** Elizabeth A. Miles, Tero Vahlberg, Philip C. Calder, Noora Houttu, Lotta Pajunen, Ella Koivuniemi, Kati Mokkala, Kirsi Laitinen

**Affiliations:** 1 School of Human Development and Health, Faculty of Medicine, University of Southampton, IDS Building, Mailpoint 887 Southampton General Hospital, Southampton, SO16 6YD UK; 2 Department of Clinical Medicine, Biostatistics, University of Turku, 20520 Turku, Finland; 3 NIHR Southampton Biomedical Research Centre, University Hospital Southampton NHS Foundation Trust and University of Southampton, Southampton, SO16 6YD UK; 4 Institute of Biomedicine, Integrative Physiology and Pharmacology, University of Turku, 20520 Turku, Finland; 5 Department of Obstetrics and Gynecology, Turku University Hospital, 20500 Turku, Finland

**Keywords:** Iodine status, Pregnancy, Infant, Urinary iodine concentration, Iodine intake

## Abstract

**Purpose:**

Iodine insufficiency during pregnancy may adversely influence fetal growth and development. There is a lack of information on iodine status in pregnant women and infants in many countries including Finland. The aim of this study is to determine dietary intake of iodine and the iodine status in a population of Finnish pregnant women and their infants.

**Methods:**

Urine samples were collected from women participating in a mother–child clinical study at early (*n* = 174) and late pregnancy (*n* = 186) and at three months of postpartum (*n* = 197), when infant samples were also collected (*n* = 123). Urine iodine concentration was measured using inductively coupled plasma mass spectrometry. Cutoffs for iodine insufficiency were < 150 µg/L during pregnancy and < 100 µg/L at postpartum and in infants. Iodine intake was assessed using 3-day food diaries.

**Results:**

Increased risk of insufficiency, based on urinary iodine concentrations, was observed in the groups investigated in this study. Of the women studied, 66% had urinary iodine concentrations indicating insufficient intakes and iodine insufficiency at early pregnancy, 70% at late pregnancy and 59% at three months of postpartum. This was also the case in 29% of the three-month-old infants. Estimation of iodine intake revealed that iodine insufficient women had lower intakes of iodine from the diet, from food supplements and from diet plus supplements than iodine sufficient women in early pregnancy and at three months of post-partum. In late pregnancy, this difference was seen for iodine intake from supplements.

**Conclusion:**

The majority of the women manifested with low urine iodine concentrations both during and after pregnancy. Similarly, one-third of the infants presented with iodine insufficiency. Maternal iodine intake data support these findings. These observations may have implications for optimal child cognitive development.

## Introduction

Iodine is an essential micronutrient required for the biosynthesis of thyroid hormones and for normal neurodevelopment during early life [1]. These roles result in an increased requirement for iodine during pregnancy to support maternal adaptation and to supply the fetus with thyroid hormones in early pregnancy and with iodine in later pregnancy [2]. Higher iodine requirement during pregnancy is recognised in guidance recommending an increased intake for pregnant women by organisations including the WHO [3]. Urinary iodine concentration (UIC) is an effective biochemical indicator to assess iodine status at the population level, with a median UIC of < 150 µg/L indicating iodine insufficiency during pregnancy and < 100 µg/L during breastfeeding and in general adult populations [3, 4].

Severe iodine deficiency during fetal development causes cognitive and neurological impairment resulting in mental retardation, learning difficulties, and mobility problems [5]. Other studies have reported that iodine insufficiency impacts child development, with children born to mothers who had iodine insufficiency during pregnancy having lower verbal IQ scores and reading comprehension at 9 years of age, and a reduced spelling score at 9 years of age [6, 7].

Many countries, particularly those in the developing world, have introduced mandatory iodine fortification of food to address the issue of dietary iodine supply [8]. In the developed world, mandatory fortification of food with iodine is less common. Zimmermann in his report of iodine status in industrialised countries reported that ‘there are insufficient data from the majority of the countries to estimate the prevalence of iodine deficiency in pregnant women’ [9]. The Iodine Global Network records that the adult population in Finland has an insufficient iodine status [8]. The 2017 National FinDiet Survey reported the median UIC for Finnish adults aged 24–74 years to be 96 µg/L indicating that much of the adult population was iodine insufficient [10]. Currently, there is a lack of data establishing the iodine status of pregnant women and infants in Scandinavia and particularly in Finland [11]. Therefore, the aim of this study is to provide data on the iodine status of a population of Finnish pregnant women and their infants. Participants were recruited to the Fish Oil and Probiotics in Pregnancy study (FOPP) which investigated the effect of fish oil and/or probiotic consumption in pregnancy on the risk of developing gestational diabetes [12].

## Materials and methods

### Study design and participants

Details of the FOPP study design and methods have been previously described [12]. Briefly, overweight and obese women with pre-pregnancy BMI ≥ 25 kg/m^2^, pregnant < 18 weeks, singleton pregnancy, and absence of chronic metabolic and gastrointestinal diseases including diabetes and inflammatory bowel disease were recruited between October 2013 and July 2017 (ClinicalTrials.gov, NCT01922791). Women (*n* = 439) were randomized to intervention groups receiving fish oil + placebo, probiotics + placebo, fish oil + probiotics or placebo + placebo at early pregnancy, the baseline of the study, and followed thereafter. The intervention with dietary supplements administered to mothers (fish oil containing 2.4 g of *n*-3 polyunsaturated fatty acids, Croda Europe Ltd, Leek, U.K., and/or probiotics *Lactobacillus rhamnosus* HN001 and *Bifidobacterium animalis ssp. lactis* 420, each 10^10^ colony-forming units) was not expected to influence the iodine status of the mothers or their infants but was nevertheless considered in the statistical analyses. Urine samples were collected from mothers at early and late pregnancy and from mothers and their infants at three months after delivery. Women who provided a urine sample at any time point and were not receiving thyroxin treatment were included in this analysis. This yielded a final sample of 174 women in early pregnancy, 186 in late pregnancy and 197 after delivery, and 123 infant samples. Details on clinical characteristics of the participants were collected by interviews and questionnaires.

### Ethics

This study was conducted according to the guidelines of the Declaration of Helsinki as revised in 2013 and the protocol was approved by the Ethics Committee of the Hospital District of Southwest Finland (115/180/2012). Written informed consent was obtained from all mothers. Ethical approval for the analysis of urinary iodine and creatinine concentrations, which were conducted at the University Hospital Southampton, United Kingdom, was additionally obtained from the East Midlands—Leicester South Research Ethics Committee (18/EM/0285).

### Urine sample collection

Mother’s urine samples were collected at early (mean (SD) 14.0 (1.8) weeks of gestation) and late pregnancy (35.2 (0.8) weeks of gestation) and from mothers and their infants at three months after delivery [child’s decimal age, mean (SD) 0.25 (0.02) years]. Mothers were instructed to wash the area of urinary meatus, dry the skin and to collect a mid-stream urine sample in sterile pots during the study visits. The time (am or pm) of sample collection, fasting status and having a drink (within two hours prior to sampling) were documented. Infant’s urine samples were taken on the morning or the previous evening of the study visit at home. Mothers were instructed to wash the area of urinary meatus of the baby with warm water or gauze wipe and to dry the skin. Special urine collection bags were used for the collection of infant samples (100 mL sterile Pediatric Urine Collector (MDS190510) by MedlLine Industries, Mundelain, Illinois, US). The urine was pipetted from the pots and collection bags to sterile tubes by a researcher, and aliquots of urine were frozen initially at − 20 °C and then stored at − 80 °C until analyses.

### Measurement of iodine status

Urinary iodine and creatinine concentrations were measured by the Trace Element Unit at the University Hospital Southampton NHS Foundation Trust. Urinary creatinine concentration was measured using the Jaffe reaction with a Beckman Coulter AU5800 clinical chemical analyser. UIC was measured in duplicate using inductively coupled plasma mass spectrometry (NexION 300D, PerkinElmer). For the urinary iodine measurements, rhodium was used as an internal standard (VWR International). Samples were analysed against potassium iodide urine standards at 0, 1, 2, 5, and 10 µmol/L (Fisher Chemicals). Urine samples were diluted 1:15 with diluent containing 1.2 g/L ammonium dihydrogen orthophosphate, 0.4 g/L ethylene-diamine-tetra-acetic acid disodium salt dehydrate, and 0.3% ammonia (Fisher Chemicals). The accuracy of the iodine analysis was verified using certified urinary iodine reference material: Seronorm trace elements urine 1 (0.83 µmol/L; range 0.66—0.99 µmol/L) and Seronorm trace elements urine 2 (2.30 µmol/L; range 1.90–2.80 µmol/L) (Sero, Norway). All measurements of the reference materials for all runs (*n* = 59) fell within the acceptable range with a percent bias of -4.2% for Seronorm trace elements urine 1 and 1.3% for Seronorm trace elements urine 2. The Trace Element laboratory participates in 2 external quality assurance schemes run by Quebec Toxicology Centre. In 2019 (the year in which the measurements were made for this study), the laboratory obtained an overall score of 93% for urinary iodine measurement in the Interlaboratory Comparison Program for metals in Biological Matrices and 96% in the Quebec Multielement Quality Assessment Scheme. Between run precision for the 2 standards gave a coefficient of variation of 4.7% and 4.2%, respectively, and within run precision coefficient of variation was 2.17% and 1.21%, respectively. UIC cutoffs for iodine insufficiency were < 150 µg/L during pregnancy and < 100 µg/L at postpartum and in infants [4].

### Estimation of iodine intake

Iodine intake was estimated from three-day food diaries (2 weekdays and 1 weekend day) recorded during the week preceding the urine sample collection. Participants were given oral and written instructions on how to fill in the food diary, including recording use of food supplements, and diaries were checked for completeness and accuracy with the help of an illustrated portion booklet. Iodine intake was calculated using computerized software (AivoDiet 2.0.2.3; Aivo, Turku, Finland) and the food composition database provided by the Finnish National Institute for Health and Welfare [13] www.fineli.fi. Intake of iodine from food supplements recorded in the food diary was calculated using manufacturer’s information.

The quality of mothers overall diet was assessed by the validated index of diet quality (IDQ) questionnaire [14] that reflects adherence to dietary recommendations (Nordic Nutrition Recommendations). This questionnaire contains 18 questions regarding the frequency and amount of consumption of foods during the preceding week (e.g. whole grains, fats including spreads and salad dressing, fish, dairy, vegetables, fruits and berries, fruit juices, sugar-containing soft drinks, sweets, and chocolate). The quality of the diet was defined as poor when index points were less than ten out of the maximum 15 points and good when points were 10 or more [14].

### Statistical analysis

Maternal and infant UIC and iodine-to-creatinine ratio distributions were positively skewed and were analyzed using non-parametric methods. The effect of intervention (fish oil and/or probiotics) on UIC and iodine-to-creatinine ratio was examined using Kruskal–Wallis test, and no effect was seen. The associations of categorical and continuous clinical characteristics with UIC and iodine-to-creatinine ratio were analyzed with using the Mann–Whitney *U* test and Spearman’s correlation coefficient, respectively. The correlation between maternal and child urine iodine concentrations was assessed using the Spearman’s correlation coefficient. Differences between iodine insufficient and sufficient women were tested using two-sample t test for intakes of iodine from diet and diet + food supplements and using Mann–Whitney *U* test for intakes of iodine from food supplements. The association of categorized dietary quality index score (good vs. poor) with UIC and iodine was examined with Mann–Whitney *U* test. Correlations of dietary quality index score and intake of iodine with UIC and iodine-to-creatinine ratio were assessed using the Spearman’s correlation coefficient. In addition, Spearman’s partial correlations between intakes of iodine and UIC and iodine-to-creatinine ratio after adjustment for sampling year, sampling time of the day (am/pm) and whether mother had a drink within two hours prior to urine sampling were conducted for the early pregnancy time point as initial analyses indicated their potential impact on urine iodine results. *P* values less than 0.05 were considered as statistically significant. Statistical analyses were performed using SAS System for Windows, version 9.4 (SAS Institute Inc., Cary, NC).

## Results

### Clinical characteristics

The clinical characteristics of the women and their infants are presented in Table 1. None of these characteristics was related to maternal or infant UIC or urinary iodine-to-creatinine ratios (all NS), except that mothers of girl babies (*n* = 80) compared to those of boy babies (*n* = 72) had higher iodine-to-creatinine ratios in early pregnancy (median (IQR) 87.4 (80.1) µg/L vs 74.5 (34.6) µg/L, *p* = 0.023), and infants of mothers with higher education (median (IQR) 152.3 (145.0) µg/L, *n* = 83) had higher UIC than those of mothers with lower education 125.5 (89.3) µg/L, *n* = 40, *p* = 0.034).

**Table 1:** Clinical characteristics of mothers and their infants. Data are reported as mean (SD), unless otherwise stated

	*N*	Mean	SD
Mothers			
Age, yrs	238	30.9	4.5
College or university degree, % (n/N)	217	64.5	(140/217)
Pre-pregnancy BMI, kg/m^2^	238	29.7	4.1
Overweight, % (n/N)	238	61.8	(147/238)
Obese, % (n/N)	238	38.2	(91/238)
Primipara, % (n/N)	238	51.3	(122/238)
GDM diagnosis in pregnancy, % (n/N)	224	32.6	73/224
Smoking during pregnancy, % (n/N)	215	8.0	(19/215)
Infants			
Sex, male, % (n/N)	123	62.2	(77/123)
Birth weight, g	123	3648	510
Birth weight, SD score	123	0.15	1.15
Weight for height at 3 mo, SD score	123	0.31	0.92
Length at 3 mo, SD score	123	− 0.36	1.12
Preterm, % (n/N)	123	4.1	(4/123)
Cord blood thyrotropin, mU/L, Median (IQR)	118	8.7	(8.2)

Most (85%, 105/123) mothers were breastfeeding their infants at 3 months of postpartum. Maternal breastfeeding status (yes/no) was not related to child’s UIC (breast-fed 137.1 (122.8) µg/L, non-breast-fed 146.6 (82.8) µg/L, *p* = 0.713) or iodine-to-creatinine ratio (breast-fed 1448.0 (915.0) µg/g, non-breast-fed 1742.9 (930.2) µg/g, *p* = 0.103). Similarly, mother’s breastfeeding status (87%, 172/197 breast-feeding) was not reflected in mother’s UIC (breast-feeding 90.0 (78.3) µg/L, non-breast-feeding 80.3 (71.0) µg/L, *p* = 0.520) or iodine-to-creatinine ratio (breast-feeding 103.0 (97.8) µg/g, non-breast-feeding 102.4 (92.1) µg/g, *p* = 0.854).

### Iodine status

UIC and iodine-to-creatinine ratio for all women at each time point, the infants and for women with iodine sufficiency and insufficiency are presented in Table 2. Of the women, 66% (114/174) were iodine insufficient at early pregnancy, 70% (130/186) at late pregnancy (UIC < 150 µg/L) and 59% (117/197) at three months of postpartum (UIC < 100 µg/L). Almost one-third (29%; 36/123) of the infants were iodine insufficient at the age of three months (UIC < 100 µg/L).

**Table 2:** Urine iodine concentration (UIC) and iodine:creatinine ratio for all and for iodine insufficient and sufficient women at early and late pregnancy and three months of postpartum and for infants at three months of age

	N^1^	All	Insufficient	Sufficient
UIC (µg/L)				
Mothers				
Early pregnancy	(174/114/60)	114.8 (107.4)	93.2 (49.8)	213.8 (123.4)
Late pregnancy	(186/130/56)	119.5 (77.3)	99.3 (52.0)	200.2 (75.2)
3 mo postpartum	(197/117/80)	88.6 (78.9)	54.8 (46.9)	141.8 (92.5)
Infants, 3 mo of age	(123/36/87)	137.7 (121.8)	70.9 (34.5)	182.1 (126.9)
Iodine:Creatinine (µg/g)				
Mothers				
Early pregnancy	(174/114/60)	79.3 (69.6)	68.5 (36.3)	123.0 (95.0)
Late pregnancy	(186/130/56)	98.3 (68.1)	85.7 (58.7)	122.2 (81.3)
3 mo postpartum	(197/117/80)	102.7 (92.7)	94.8 (69.0)	122.6 (123.4)
Infants, 3 mo of age	(123/36/87)	1502.0 (933.9)	1101.5 (762.1)	1626.7 (902.6)

Data are reported as median (IQR)

^1^*N* for all, insufficient and sufficient groups

### Correlation of maternal and child urine iodine concentration

There was no significant correlation between maternal UIC at any time point and infant UIC (all NS). Maternal iodine-to-creatinine ratio correlated at late pregnancy (*r* = 0.21, *p* = 0.041) and at 3 months of postpartum (*r* = 0.25, *p* = 0.006), but not at early pregnancy (*r* = 0.20, *p* = 0.082), with infant’s values.

### Dietary intake of iodine in relation to iodine status

Iodine insufficient women had lower intakes of iodine from the diet, from supplements and from diet + supplements than iodine sufficient women in early pregnancy and 3 months of postpartum (Table 3). In late pregnancy, this difference was only seen for iodine intake from supplements (Table 3). Furthermore, maternal use of multivitamin supplements (71% (124/174 of the women) at early pregnancy, 66% (123/186) at late pregnancy and 53% (103/196) at postpartum) was related to higher maternal UIC (data not shown). Also, maternal use of multivitamin supplements at postpartum (54%, 66/122) was related to child UIC (176.6 vs 144.8 µg/g in users vs. non-users, *p* = 0.029, Mann–Whitney *U* test).

**Table 3:** Intake of iodine from diet, food supplements and diet + food supplements (total) in all women and in those with iodine insufficiency and sufficiency at early and late pregnancy and at three months of postpartum

	N^1^	All	Iodine intake (μg/day)		*p* value*
			Insufficient	Sufficient	
Early pregnancy	(167/109/58)				
Diet, Mean (SD)		191 (59)	182 (54)	208 (65)	0.006
Food supplements, Median (IQR)		140 (150)	117 (140)	140 (58)	0.0004
Total, Mean (SD)		290 (94)	266 (90)	335 (85)	< 0.0001
Late pregnancy	(176/122/54)				
Diet, Mean (SD)		205 (63)	203 (66)	208 (57)	0.669
Food supplements, Median (IQR)		140 (175)	117 (150)	140 (175)	0.023
Total, Mean (SD)		301 (110)	292 (113)	321 (100)	0.112
3 mo postpartum	(180/105/75)				
Diet, Mean (SD)		206 (70)	197 (64)	219 (77)	0.034
Food supplements, Median (IQR)		75 (175)	0 (145)	140 (175)	0.022
Total, Mean (SD)		285 (112)	263 (105)	316 (115)	0.002

Data are reported as mean (SD) or median (IQR)

*Statistically significant difference between iodine insufficient and sufficient women; *t* test for intakes from diet and diet + food supplements, Mann–Whitney *U* test for intake from the food supplements

^1^*N* for all, insufficient and sufficient groups

Maternal dietary quality index score (mean (SD) at early pregnancy 9.4 (2.9), at late pregnancy 9.7 (1.9) and at postpartum 9.4 (2.1)) did not correlate with UIC at any of the time points (early pregnancy: *r* = 0.05, *p* = 0.477, *n* = 173; late pregnancy: *r* = 0.07, *p* = 0.343, *n* = 184 and postpartum: *r* = 0.12 *p* = 0.103, *n* = 195). Iodine-to-creatinine ratio was found to correlate with dietary quality index score at early pregnancy (*r* = 0.18, *p* = 0.016) and at postpartum (*r* = 0.25, *p* = 0.0005), but not at late pregnancy (*r* = 0.11, *p* = 0.132).

A correlation was found between the dietary quality index score at early pregnancy (*r* = 0.23, *p* = 0.046, *n* = 79), but not at late pregnancy (*r* = 0.17, *p* = 0.088, *n* = 97) or at postpartum (*r* = 0.15, *p* = 0.092, *n* = 122) with infants’ UIC. Similarly, considering infant’s iodine-to-creatinine ratio, correlations with maternal dietary quality index score were seen at early pregnancy (*r* = 0.230, *p* = 0.042), but not at late pregnancy (*r* = 0.01, *p* = 0.937) or at 3-month postpartum (*r* = 0.03, *p* = 0.740).

Using the categorized dietary quality score, a higher urine iodine-to-creatinine ratio was seen in mothers with good diet quality compared to those with poor diet quality at early pregnancy (median (IQR) 87.7 (81.9) and 74.8 (44.4), respectively, *p* = 0.047) and at postpartum 113.0 ((117.6) and 91.5 (72.3), *p* = 0.006), but not at late pregnancy (104.0 (67.5) and 93.0 (70.9), respectively, *p* = 0.107) or with infants’ urine iodine values (all NS).

Intake of iodine from the diet correlated weakly with UIC at early pregnancy (*r* = 0.20, *p* = 0.009, *n* = 167), at late pregnancy (*r* = 0.16, *p* = 0.031, *n* = 176) and postpartum (*r* = 0.16, *p* = 0.034, *n* = 180). A stronger correlation was detected for iodine intake from supplements at each time point (early pregnancy: *r* = 0.27, *p* = 0.0005; late pregnancy: *r* = 0.32, *p* < 0001; postpartum: *r* = 0.24, *p* = 0.001), as well as for total dietary intake of iodine from diet plus supplements (early pregnancy: *r* = 0.34, *p* < 0.0001; late pregnancy: *r* = 0.34, p < 0.0001; postpartum: *r* = 0.29, *p* < 0.0001).

Iodine-to-creatinine ratio correlated with dietary intake at early pregnancy (*r* = 0.18, *p* = 0.02, *n* = 167) and postpartum (*r* = 0.24, *p* = 0.001, *n* = 180), but not at late pregnancy (*r* = 0.10, *p* = 0.173, *n* = 176). For supplements and total (diet + supplements) intakes, correlations with iodine-to-creatinine ratio were seen at early (*r* = 0.29, *p* = 0.0001 and *r* = 0.36, *p* < 0.0001, respectively, *n* = 167) and late (*r* = 0.38, *p* < 0.0001; *r* = 0.36, *p* < 0.0001, *n* = 176) pregnancy and postpartum (*r* = 0.44, *p* < 0.0001, *n* = 179; *r* = 0.47, *p* < 0.0001), *n* = 180).

The results for the early pregnancy time point remained essentially the same regardless of the adjustment for sampling year, sampling time of the day (am/pm) and whether the mother had a drink within two hours prior to urine sampling.

## Discussion

In this study, we demonstrated using WHO criteria that 60–70% of the mothers were iodine insufficient during and after pregnancy. Further, 29% of the infants were iodine insufficient (UIC < 100 ug/L) at 3 months of age. These are the first data reporting iodine status in Finnish pregnant women and infants. Our data indicate that iodine insufficiency is present in pregnancy and postpartum and in infants which may have implications for optimal cognitive development in the children. There are data demonstrating that children born to mothers with either low UIC or dietary iodine intakes have a greater risk of poorer development including lower cognitive, language, and motor scores and cognitive development delay [6, 7, 15].

Pregnant women consumed a mean intake of 191, 205 and 206 ug/d iodine from food and 99, 96 and 79 ug/d from supplements, and 290, 301 and 285 ug/d (early pregnancy, late pregnancy and postpartum) from food plus supplements. These intake data are in agreement with national diet survey data from FINDIET 2017 showing intakes of iodine in Finnish women of 192 ug/d from food, 81 ug/d from supplements, with a total intake of 273 ug/d for supplement users and 184 ug/d for those not using supplements [10]. In the current study, the mean dietary intake of iodine from food without supplements falls below the WHO recommended intake of iodine for pregnant women of 250 ug/d. Maternal diet quality at early pregnancy was also correlated with both UIC and urinary iodine creatinine ratio in the infants.

Maternal iodine intake from the diet showed a weak correlation with maternal ante and postpartum UIC. A rather stronger correlation was seen between maternal iodine intake from supplements and UIC and for combined dietary and supplement intake of iodine in the mothers ante and postpartum. This is in keeping with results reported from studies of iodine in pregnant women in other countries [16–18]. Estimating iodine intake is a challenge [19]. Food iodine content depends on many factors including how and where food is grown (iodine content of soil, use of fertiliser, and addition of iodine to animal feeds) and processing (e.g. fortification including addition of iodised salt) [20, 21]. Iodised salt can be a significant dietary source of iodine but is difficult to estimate by a food frequency questionnaire or even weighed portion food diary. The iodine intake in this study suggests that the women have an intake which is close to sufficient, and this is in agreement with the FINDIET iodine intake data [10]. However, again in agreement with data generated for the large FINDIET 2017 cohort (*n* = 1542), the UIC suggests that a substantial proportion of the population is at risk of being iodine insufficient [22]. Interestingly, the FINDIET report comments that the discrepancy between the dietary intake data and the UIC data for the FINDIET data may be partially explained by discrepancies in the food database which calculates food iodine content based upon the constituent foods rather than the iodine content of prepared foods [10]. Food databases may also not be up to date regarding the use of iodised salt vs non-iodised salt in food production and in home cooking. Similarly, we suggest that the dietary intake data may be overestimating actual dietary iodine intake in the current study.

The majority of infants in this study (85%) were breastfed at 3 months of age. There was no significant difference in UIC of those infants who were breastfed and those who were not. Similarly, Nazeri et al. reported no difference between the UIC for formula fed and breastfed Iranian infants of less than 3 months of age [23]. These results showing that breastfed and formula fed infants did not differ in UIC and that a larger proportion of the mothers than infants in this study fell within the insufficient range suggests that infant iodine supply is favoured over maternal status. Nonetheless, almost one-third of the infants in this study were classed as iodine insufficient.

Infant UIC was not related to maternal dietary intake of iodine. However, UIC was significantly higher for infants whose mothers used multivitamin supplements postpartum when compared with infants whose mothers did not. This is consistent with data from other studies of iodine in breastfeeding mothers and their infants [24].

Finland has a history of iodine deficiency indicated by a high prevalence of goitre endemic within the population [25]. Changes in Finnish farming practice along with a successful policy for the use of iodised salt addressed the shortfall in iodine intake in the Finnish population [25]. However, more recently there has been concern that the iodine intake and status of the population in Finland may have decreased. The national diet survey in Finland data showed that the average adult intake of iodine was 117 ug per day which does not meet the WHO recommended intake of 150 ug per day for adults and 250 ug per day for pregnant and lactating women [3, 26]. This raised concern and resulted in the introduction of the use of iodised salt in food manufacturing in Finland [27]. Although these actions have improved the iodine status [10] which has been reflected in an increase in the UIC for adults in data obtained for samples collected 2012 and 2017 [22], some individuals are likely to be iodine insufficient and the reported adult population UIC for Finland is lower than for most other Nordic countries [8, 22]. Our data on Finnish pregnant women and infants demonstrate iodine insufficiency in a considerable proportion of the populations which are most sensitive to iodine deficiency.

This study has several limitations including the sample size, the selection of the study population, and the use of spot urine samples.

The lack of data available for the iodine status of pregnant women and children in Finland was the reason that we chose to measure iodine in the samples from the FOPP study. The cohort was not large (*n* = 197), and the women recruited to the FOPP study were generally healthy pregnant women who were overweight or obese and randomized to take probiotic and fish oil study supplements during their pregnancy. Thus, the BMI status meant that the study population may not be representative of the general population of pregnant women and that the intervention needed to be considered in the data analysis.

Within our cohort, body mass index (BMI) was not associated with iodine intake or UIC in the mother nor UIC in the infants. Knight et al. in a cohort of pregnant women in the UK who were not selected for a higher BMI (median and interquartile range 24.4, 22.0–28.3 kg/m^2^) similarly report no association of UIC with BMI and this was also seen in a cohort of pregnant women with obesity in the UK [28, 29]. However, there are studies which have seen a negative association between UIC in non-pregnant morbidly obese women and BMI [30]. In addition, the study intervention (fish oil and probiotic supplements) did not significantly affect the outcomes measured in this study.

The use of spot urine samples rather than a 24 h urine collection was also a limitation. Therefore, we measured creatinine in the samples to correct for urinary dilution and expressed the data as the iodine:creatinine ratio. However, the use of creatinine to correct for urine volume is not without issue, particularly in infants where a low muscle mass results in a low creatinine excretion and a higher iodine:creatinine ratio [31]. For these reasons, we have reported our data as both UIC and iodine:creatinine ratio.

The strengths of this study are that it is addressing the current lack of data regarding iodine status in those in the population most vulnerable to effects of iodine deficiency. To our knowledge, this is the first mother–baby paired iodine status data for Finland. The cohort was similar to the general population of pregnant women in Finland in 2015 with regard to maternal age (mean age in this study 30.9 years vs. perinatal statistics 30.6 years) and delivery parameters but the proportion of primipara women was slightly lower in our sample (51.3 vs 58.4%) [32, 33]. The cohort is well characterised and we have collected both intake and status data. Data have been collected for the mothers during pregnancy and postpartum and their infants at three months of age. The methodology used is robust: UIC was measured in duplicate using inductively coupled plasma mass spectrometry (the gold standard for this analysis) in an internationally recognised laboratory who have previously analysed urinary iodine in large cohort studies [6, 34]. The dietary intake data are in agreement with the latest national survey data for iodine intake for adults in Finland [10].

## Conclusion

Our study has provided evidence to demonstrate that according to UIC, a significant proportion of women in Finland are at risk of being iodine insufficient during pregnancy and that around a third of their infants also fall into the insufficient category as defined by WHO [4]. This is supported by data showing a suboptimal iodine intake in the mothers during pregnancy and postpartum. This may have implications for the optimal cognitive development of children.

Recommendations were made in Finland in 2015 to improve iodine status through the use of iodized salt in food industry, but data from our study suggest that this had not resulted in iodine sufficiency at least in the most vulnerable groups within the population.

Measuring the iodine status in a larger cohort of pregnant women and of children in Finland should be done to establish whether further public health measures are required to achieve iodine sufficiency within the most vulnerable sectors of the population.

## Abbreviations

BMI: Body mass index
FOPP: Fish oil and probiotics in pregnancy study
UIC: Urinary iodine concentration
